# A Plague Report from Sydney

**Published:** 1909-02-27

**Authors:** 


### Tropical Diseases.

### A PLAGUE REPORT FROM SYDNEY.

Some years ago Sydney was unfortunate enough to become infected with plague, and since that time numerous new outbursts or reinfections have taken place. Dr. Ashburton Thompson, who has studied these in great detail, now reports on a seventh outbreak in 1907 and Dr. R. J. Millard on an outbreak at Kempsey on the Macleay River during the same year.* The epidemiological features of these small epidemics have been so ably described by the two authors above mentioned that they form most interesting reading. The association of plague and infected rats is clear for most of the Sydney cases, and quite probably such an association would always be found in other countries if looked for as carefully as has been done in Sydney.

The recent Plague Research Committee in India revived the old idea of the flea being the intermediary between the rat and man, and they not only revived the idea but also proved it, at least to the satisfaction of most people. Dr. Thompson from his work on the previous outbreaks in Sydney came to very much the same conclusion, and in this connection a quotation of paragraph 22 of his present report will not be out of place: "22. The flea, acting as a living intermediary, alone remains. A preliminary question in relation to this channel is whether the evidence which excludes deposited infection as a cause is not of such a nature as to exclude also communication from man to man by the flea; but the conditions necessary to enable the flea to act as a carrier from man to man are not frequently fulfilled in civilised life. The disease in the rat is almost always a septicæmia, it is almost always fatal, and that animal carries many fleas as long as illness lasts. But the contrary is the case with man in this country. Usually he and his dwelling have not many fleas; and usually the disease does not assume the septicæmic form with him, or does so as a rule only for a short time before death when, most often, patients have already been removed to hospital. If, however, man be infested with fleas, if his disease do assume the septicæmic form, and if he do die at home, then fleas which bite him are likely occasionally to receive the infection from him, and, as has now been experimentally shown, can retain their power of communicating it during several days. This I inferred in 1902 must be the case (see Report on the second outbreak, dated 31st July, 1903, par. 300). The necessary conditions were fulfilled in these groups." It may be taken as certain now, that leaving out direct contact cases such as plague pneumonia, infection comes to man by the bites of fleas that have been living on and imbibing the blood of infected rats. Avoid being bitten by fleas—and this will include of course the keeping out of rats from dwelling-houses—and there should be no danger of acquiring plague. This will come in time, but for countries such as India it will be expensive and somewhat difficult of accomplishment. Other interesting matters contained in the Report are the experiments on the destruction of rats by "Azoa" and by "Rattin" by Dr. Millard. Recently these and other similar preparations have been boomed commercially, but it is evident that they have only a limited use and will not destroy all the given rats of any definite area. Dr. Millard's work supports this very strongly and recently Handson and Williams in London have shown that the use of some of them may even be dangerous to human beings. Haffkine in a recent paper read before the Epidemiological Society of London puts the matter in a nut-shell, thus: "Regarding this latter point (*e.g.* the spreading amongst rats of artificial epizöotics by Danysz's rat virus), one cannot forget that India has now had, in the plague bacillus, 11 years' experience of a most devastating virus for these animals, and as yet there is no sign that this involuntary experiment has rid the country of their presence."

All who have the time and are sufficiently interested in the subject of plague should read this most instructive Report for themselves.

* 1903. (Second Session) Legislative Assembly, New South Wales. Report of the Board of Health on Plague in New South Wales, 1907. On a seventh outbreak of plague at Sydney, 1907. By J. Ashburton Thompson, M.D., D.P.H., President, Chief Medical Officer of Government.
On an outbreak of plague at Kempsey, Macleay River, 1907. By R. J. Millard, M.B., Ch.M. (Syd.), D.P.H. (Camb.), Assistant Medical Officer of Government and Acting Microbiologist. Printed under No. 9 Report from Printing Committee, October 15, 1908. Sydney: William Applegate Gullick, Government Printer. 1908. 2s. 6d.

